# Estrogen Related Receptor Alpha (ERRα) a Bridge between Metabolism and Adrenocortical Cancer Progression

**DOI:** 10.3390/cancers14163885

**Published:** 2022-08-11

**Authors:** Paola Avena, Arianna De Luca, Adele Chimento, Marta Claudia Nocito, Sara Sculco, Davide La Padula, Lucia Zavaglia, Matteo Giulietti, Constanze Hantel, Rosa Sirianni, Ivan Casaburi, Vincenzo Pezzi

**Affiliations:** 1 Department of Pharmacy and Health and Nutritional Sciences, University of Calabria, 87036 Arcavacata di Rende, Italy; 2 Department of Specialistic Clinical and Odontostomatological Sciences, Polytechnic University of Marche, 60131 Ancona, Italy; 3 Department of Endocrinology, Diabetology and Clinical Nutrition, University Hospital Zurich and University of Zurich, 8091 Zürich, Switzerland; 4 Medizinische Klinik und Poliklinik III, University Hospital Carl Gustav Carus Dresden, 01307 Dresden, Germany

**Keywords:** adrenocortical cancer, ERRα, XCT790, cancer progression, mitochondria, metabolic changes

## Abstract

**Simple Summary:**

Adrenocortical carcinoma (ACC) is a rare and highly aggressive tumor associated with a very poor prognosis, mostly due to a high risk of recurrence and limited therapeutic options. The identification of “master regulators” of the metabolic changes occurring in cancer cells could offer new targets for innovative therapies. Such a strategy has never been used against ACC progression. In this study, we identify ERRα as key player in ACC metabolism and its targeting can prevent progression to a more aggressive phenotype. The development of new therapeutic strategies to selectively target ERRα in the adrenal with a selective antagonist would hinder ACC progression, avoiding off-target effects.

**Abstract:**

The aim of this study was to investigate the metabolic changes that occur in adrenocortical cancer (ACC) cells in response to the modulation of Estrogen Related Receptor (ERR)α expression and the impact on ACC progression. Proteomics analysis and metabolic profiling highlighted an important role for ERRα in the regulation of ACC metabolism. Stable ERRα overexpression in H295R cells promoted a better mitochondrial fitness and prompted toward a more aggressive phenotype characterized by higher Vimentin expression, enhanced cell migration and spheroids formation. By contrast, a decrease in ERRα protein levels, by molecular (short hairpin RNA) and pharmacological (inverse agonist XCT790) approaches modified the energetic status toward a low energy profile and reduced Vimentin expression and ability to form spheroids. XCT790 produced similar effects on two additional ACC cell lines, SW13 and mitotane-resistant MUC-1 cells. Our findings show that ERRα is able to modulate the metabolic profile of ACC cells, and its inhibition can strongly prevent the growth of mitotane-resistant ACC cells and the progression of ACC cell models to a highly migratory phenotype. Consequently, ERRα can be considered an important target for the design of new therapeutic strategies to fight ACC progression.

## 1. Introduction

Adrenocortical carcinomas (ACC) are rare and highly aggressive tumors, associated with a very poor prognosis, mostly due to a high risk of recurrence and limited therapeutic options [1]. Currently, surgery and adjuvant treatment with the adrenolytic drug mitotane, alone or in combination with chemotherapeutics, represent the only therapeutic approaches, which are very often ineffective [2]. Thus, the widening of our knowledge on the molecular pathways involved in ACC biology represents a necessary step to develop more effective and specific treatment options. Genomic characterizations of ACC identified a correlation between tumor onset and several genetic mutations [3,4], revealing high heterogeneity and histotype-specific genomic profiles [5]. These studies confirmed that ACC progression relies on a large number of potentially targetable molecules and signaling pathways, including those associated with a profound reprogramming of cellular metabolism [6].

The identification of “master regulators” of the metabolic changes would help in defining new targets for innovative therapies. This strategy has never been used to counteract ACC progression. During the last decade, members of the nuclear receptors estrogen-related receptors (ERRs) family and the peroxisome proliferator activated receptor γ (PPARγ)-coactivator-1s (PGC-1s), have been shown to work in concert to regulate mitochondrial biogenesis and metabolic pathways [7]. The ERR alpha (ERRα) subtype, controls energy homeostasis in both physiological and pathological conditions [8]. ERRα is an adopted orphan receptor since the discovery of cholesterol as its endogenous ligand [9]. Cholesterol increases PGC-1s recruitment to ERRα and up-regulates its transcriptional activity [10,11]. ERRα overexpression has been discovered in several cancerous tissues, including breast [12], ovary [13], prostate [14] and colon [15], and is associated with more aggressive behavior, suggesting that its expression can act as a prognostic marker for hormone-related tumors. Moreover, ERRα and its co-activators have been found to be involved in tumor cell motility and metastasis [16,17].

Metastasizing cells undergo dynamic metabolic changes to successfully adapt to the variations in signaling pathways and global gene expression programs that drive the metastatic cascade, including epithelial to mesenchymal transition (EMT) [18]. In this scenario, metabolic reprogramming driven by ERRα could affect the metastatic potential of cancer cells. Starting from these observations, the aim of this study was to investigate the metabolic changes that occur in ACC cells in response to the modulation of ERRα expression and the involvement of this metabolic regulator in ACC progression.

## 2. Materials and Methods

### 2.1. Cell Cultures

Adrenocortical cancer cells H295R and SW13 cells were obtained from the American Type Culture Collection (ATCC: Rockville, MD, USA). H295R were cultured in DMEM/F12 (Dulbecco’s modified Eagle’s Medium/Nutrient Mixture-F12 Ham) supplemented with 1% ITS (Insulin-Transferrin-Selenium) Liquid Media Supplement, 5% fetal bovine serum (FBS), 2.5% nu-serum, 1% glutamine, 1% penicillin/streptomycin (complete medium) while SW13 cells were maintained in DMEM/F12 supplemented with 10% FBS, 1% glutamine, 1% penicillin/streptomycin. MUC-1, the cell line isolated from a mitotane- and chemo-resistant tumor [19], were cultured in Advanced DMEM/F12 Medium containing 10% FBS and 1% penicillin/streptomycin. All cell lines were cultured at 37 °C in 5% CO_2_ in a humidified atmosphere. DMEM/F12 and supplements were purchased from Sigma (Sigma-Aldrich: Milano, Italy) while Advanced DMEM/F12 Medium and ITS were purchased from Gibco (Gibco: Thermo Fisher Scientific: Monza, Italy). Additionally, specific experiments were performed on cells grown in a medium containing lipoprotein-free serum (LpFS) (Sigma).

### 2.2. Proteomic Analysis

To identify differentially regulated proteins upon ERRα inhibitor, H295R cells, cultured in complete medium for 24 h were treated with XCT790 (10 µM) in serum free-medium (SFM) for the next 24 h while a cell plate was left in SFM and treated with DMSO (0.01%) as control. At the end of the experiment, the cells were lysed in a lysis buffer (Urea 8M in 100 mM Tris pH 8.0). Lysis was followed by two sonication cycles for a maximum time per cycle of 2 min. Samples were analyzed at the Cogentech institute in Milan. 50 µg for each sample were digested with Lys-C and trypsin, desalted C18 and injected into technical replicate in Data Dependent Acquisition (DDA) using a Q-ex-HF spectrometer, with a gradient setting equal to 75 min. Data were submitted to Maxquant for Label Free quantitative analysis against a Human database and statistical analysis was performed with Perseus tool. Finally, the ANOVA analysis was performed on three samples and then *t*-tests (*p* < 0.05) in pairs. The post-analytical phase made use of the easyGSEA tool for gene set enrichment analysis based on pathways described in Kyoto Encyclopedia of Genes and Genomes (KEGG).

### 2.3. Western Blot Analysis

Proteins were subjected to western blot analysis as previously reported [20]. Membranes were incubated overnight at 4 °C with anti-ERRα polyclonal antibody (Abcam: Cambridge, UK; dilution 1:1000) and anti-Vimentin (Santa Cruz Biotechnology, Inc.: Bergheimer Str. 89-2 69115 Heidelberg, Germany; dilution 1:1000). GAPDH antibody (Santa Cruz Biotechnology; dilution 1:2000) was used as an internal control. Membranes were incubated with horseradish peroxidase–conjugated secondary antibodies (Amersham Pharmacia Biotech: Piscataway, NJ, USA) for 1 h at room temperature. Proteins were visualized with the Western Blotting Luminol Reagent (Santa Cruz Biotechnology) and exposed to Kodak X-Omat film (Santa Cruz Biotechnology). Where indicated, the bands intensity of western blot images was measured using the NIH ImageJ software (National Institutes of Health (NIH): Bethesda, MD, USA).

### 2.4. Transfection Assays

#### 2.4.1. Stable Transfection

H295R cells were grown in a complete, antibiotic-free medium in 6 well plates (5 × 10^5^ cells/well). After 48 h, cells were transfected with DNA plasmids: a plasmid encoding a scrambled short hairpin RNAs (shRNA) sequence (shCTR) (Santa Cruz, sc108060, Dallas, TX, USA), a plasmid expressing a shRNA to inhibit ERRα gene (shERRα−/−) (Santa Cruz, sc44706-sh), according to the manufacturer’s protocol (Santa Cruz Biotecnology, https://www.scbt.com/it/resources/protocols/shrna-plasmid-dna-mediated-inhibition-of-gene-expression (accessed on 7 January 2020)), or ERRα cDNA expression plasmid(ERRα+/+) (ADGENE, #10975), according to X-tremeGENE™ HP DNA Transfection Reagent Protocol (Sigma). After 72 h, transfected cells were selected by the addition of puromycin (for shERRa−/−) or Geneticin (G418) (for ERR+/+) (10 µg/mL) (Sigma). Cells resistant to antibiotics formed clones that were isolated and amplified. In about three weeks the concentration of antibiotics was gradually decreased to 1 µg/mL.

#### 2.4.2. Transient Transfection

H295R cells were grown in complete but antibiotic-free medium in 6-well plates (5 × 10^5^ cells/well) for 48 h and then transfected with an empty vector (EV) or an ERRα plasmid expression vector (pcDNA3.1 ERRα, kindly provided by Dr. Janet E. Mertz) by X-tremeGENE™ HP DNA Transfection Reagent following manufacturer’s instructions (Sigma) for additional 48 h. After transfection, cells were treated for 24 h with XCT790 (10 μM) or grown in non-adherent conditions as 3D spheroids for 5 days.

### 2.5. Colony Formation Assay

MUC-1 cells (2 × 10^3^ cells/well) were seeded in 12-well plates and allowed to grow in the absence or presence of XCT790 (1, 5, 10 µM) for 14 days. Colonies were stained and fixed with Coomassie Brilliant Blue Solution containing methanol (Sigma) for 10 min. Colonies (>50 cells) were counted by Image J (NIH) software.

### 2.6. Wound Healing Assay

Cells were cultured in 12-well plates until approximately 80–90% confluence was achieved, then a 10 μL tip was used to create a clear-edged scratch/wound across the well width of H295R wild type (WT), *knock in* (ERRα+/+) or *knock out* (shERRα−/−) for ERRα gene and stably transfected with control plasmid (shCTR). Cells were stained and fixed with Coomassie Brilliant Blue Solution containing methanol (Sigma) for 10 min at 0 and 18 h after scratching. Photographs were acquired with 10× objective using an inverted phase contrast microscope (Olympus CKX53).

### 2.7. Boyden Chamber Assay

Cell migration was evaluated by using transwell inserts (8 μm pore size, 24-well plate, Corning Costar: Cambridge, MA, USA). Cells (4 × 10^4^/well for H295R clones, H295R and MUC-1; 10 × 10^4^/well SW13) were seeded in the insert and vehicle (DMSO) (Sigma-Aldrich: Milano, Italy), XCT790 (1, 5, 10 μM) or Mitotane (2.5, 25, 40 μM) were added in the upper chamber. Cells were allowed to migrate across the membrane for 24 h, and then stained and fixed with Coomassie Brilliant Blue Solution containing methanol (Sigma) for 10 min. Photographs (5 fields/insert) were acquired with 10× objective using an inverted phase contrast microscope (Olympus CKX53) and cells were counted by Image J (NIH) software.

### 2.8. Cell Viability Assay

The effect of XCT790 on SW13 cell viability was measured using MTT assay as previously described [21].

### 2.9. Spheroids Cultures

A single suspension of H295R clones (shCTR, shERRα−/−, ERRα+/+) and adrenocortical cells (H295R, SW13, MUC-1) were prepared using 1X Trypsin-EDTA (ethylenediaminetetraacetic acid) solution (Sigma) and manual disaggregation (21 gauge needle) [22]. Cells were seeded in non-adherent conditions, as previously described [23]. Spheroids were enriched by dissociating and reseeding cells 5 times (once a week) in non-adherent conditions, and then tested (H295R Sph-5) for Boyden Chamber Assay.

### 2.10. Seahorse Xfe96 Metabolic Flux Analysis

#### 2.10.1. ATP Rate Assay

The XF Real-Time ATP Rate Assay was determined using the Seahorse Extracellular Flux Analyzer (XFe96, Agilent Technologies Inc.: 5301 Stevens Creek Blvd. Santa Clara, CA, USA). Adrenocortical cancer cells (H295R, SW13, MUC-1) and H295R clones (shCTR, shERRα−/−, ERRα+/+) were seeded into XF96-well cell culture plates (Seahorse Bioscience, MA, USA) and incubated overnight at 37 °C in a 5% CO_2_ humidified atmosphere. After 48 h, cells were treated with XCT790 (1, 5, 10 μM) for 18 h. At the end of treatment, cells were washed in warm XF assay media supplemented with 10 mM glucose, 1 mM Pyruvate, 2 mM L-glutamine, and adjusted at pH 7.4. Cells were then maintained for 1 h in 175 μL/well of XF assay media at 37 °C, in a non-CO_2_ incubator. During the cell incubation time, 25 μL of a solution of XF assay media containing 15 µM oligomycin, 5 μM rotenone/antimycin A, were loaded into the injection ports of the XFe-96 sensor cartridge. The dataset was analyzed by XFe-96 software (Agilent).

#### 2.10.2. Mitochondrial Stress Analysis

Real-time oxygen consumption rates (OCR) were determined using the Seahorse Extracellular Flux analyzer (XF96) (Agilent). Adrenocortical cancer cells (H295R, SW13, MUC-1) and H295R clones (shCTR, shERRα−/−, ERRα+/+) were seeded into XF96-well cell culture plates (Seahorse Bioscience: North Billerica, MA, USA) and incubated overnight at 37 °C in a 5% CO_2_ humidified atmosphere. After 48 h, cells were treated with XCT790 (1, 5, 10 μM) for 18 h. At the end of treatment, cells were washed in warm XF assay media supplemented with 10 mM glucose, 1 mM Pyruvate, 2 mM L-glutamine and adjusted at pH 7.4. Cells were then maintained for 1 h in 175 μL/well of XF assay media at 37 °C, in a non-CO_2_ incubator. During the cell incubation time, 25 μL of a solution of XF assay media containing 10 μM oligomycin, 9 μM FCCP, 10 μM rotenone, 10 μM antimycin A were loaded into the injection ports of the XFe-96 sensor cartridge. The dataset was analyzed by XFe-96 software (Agilent).

#### 2.10.3. Glycolytic Stress Analysis

The extracellular acidification rate in real time (ECAR) was determined using the Seahorse Extracellular Flux Analyzer (XF96) (Agilent). Adrenocortical cancer cells (H295R, SW13, MUC-1) and H295R clones (shCTR, shERRα−/−, ERRα+/+) were seeded into XF96-well cell culture plates (Seahorse Bioscience, MA, USA), and incubated overnight at 37 °C in a 5% CO_2_ humidified atmosphere. After 48 h, cells were treated with XCT790 (1, 5, 10 μM) for 18 h. At the end of treatment, cells were washed in a specific buffer (XF medium, pH 7.4) for the determination of metabolic flows added with 2 mM of L-glutamine. The cells were then maintained for 1 h in 175 μL of XF medium at 37 °C, in an incubator without CO_2_. During the incubation time, the XF buffer solution (25 µL) containing glucose (10 mM) oligomycin (1μM), 2-deoxy-D-glucose (50 mM) was added into the injection ports. ECAR measurements were normalized to protein content within the individual wells. The dataset was analyzed by XFe-96 software (Agilent).

### 2.11. Statistical Analysis

All experiments were performed at least three times. Data are expressed as mean values ± standard deviation (SD). The statistical significance was analyzed using GraphPad Prism 5.0 software (GraphPad Soft-ware, Inc., San Diego, CA, USA). Normality was assessed using the Kolmogorov–Smirnov, D’Agostino & Pearson omnibus and Shapiro–Wilks test, with a *p* value < 0.05. When the results satisfied the normality (Gaussian distribution and equal variance), unpaired t-tests with Welch correction or ANOVA (analysis of variance) with post hoc Bonferroni test were used. When data did not meet normality, the non-parametric Mann–Whitney’s test (for independent comparisons), and Kruskal–Wallis test (for multiple comparisons) with post hoc Dunns test were used.

## 3. Results

### 3.1. Proteomic Analysis of H295R Cells: Effects of XCT790 on Cell Metabolism

**Figure 1:**
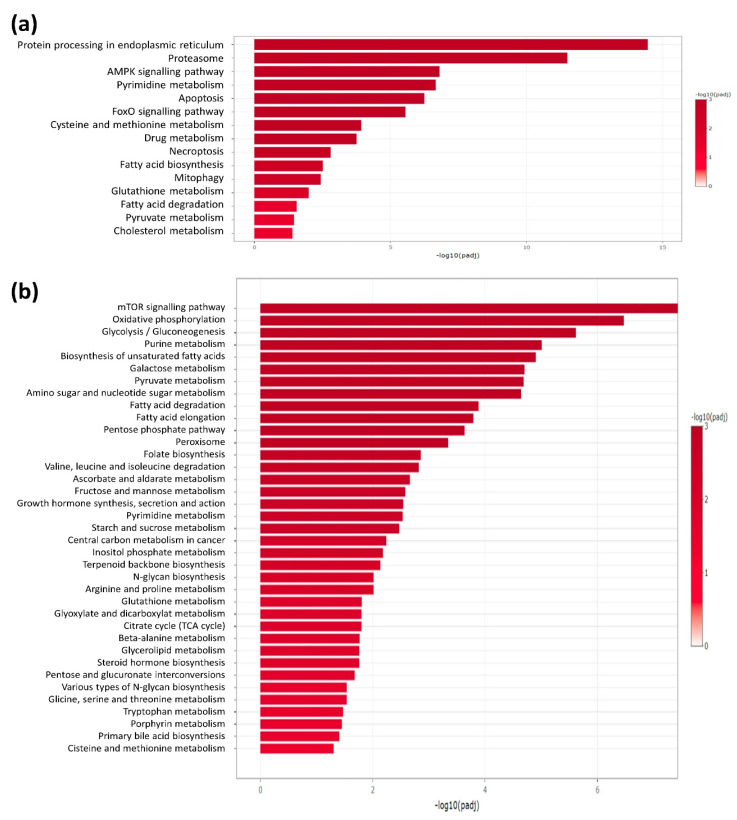
Functional enrichment analysis of differentially expressed proteins (**a**): up-regulated; (**b**): down-regulated proteins) between untreated and XCT790-treated cells. KEGG database and selected metabolism-related pathways were used. Only significant enriched pathways are reported (FDR-adjusted *p*-value, padj < 0.05, i.e., −log10(padj) > 1.3).

To identify differentially regulated proteins upon ERRα inhibitor, label-free quantitative proteomic analysis of H295R cells were performed. Differential expression analysis between untreated and XCT790-treated cells showed significant changes for a large amount of proteins. Specifically, the analysis revealed significant modulation of 1447 genes including 757 up-regulated and 690 down-regulated genes. Using the Kyoto Encyclopedia of Genes and Genomes (KEGG, https://www.genome.jp/kegg/ accessed on 2 March 2022) pathway database, we identified that these proteins fall within several pathways with significant relative abundance. As shown in Figure 1, most pathways related to cell metabolism were down-regulated by XCT790. Starting from these data we next investigated the metabolic functions of the available ACC cell models in response to ERRα manipulation.

### 3.2. Role of ERRα in Metabolic Functions of Different ACC Cell Lines

**Figure 2:**
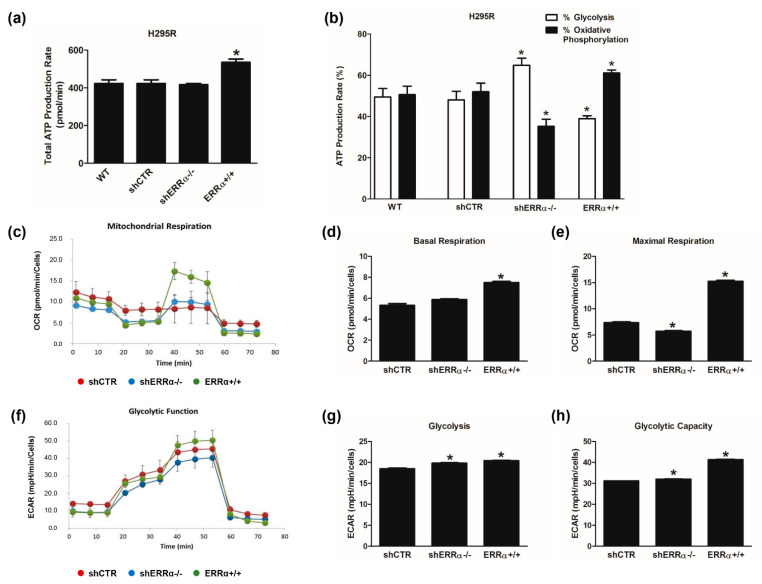
Metabolic changes in H295R cells related to ERRα expression levels. The metabolic profiles of H295R wild type (WT), shCTR, shERRα−/− and ERRα+/+ cells were assessed by Seahorse XFe96 Analyzer. (**a**,**b**) ATP Rate Assay was evaluated as indicated in “Materials and Methods”. Graphs represent the mean ± SD of three independent experiments of Total ATP Production Rate (pmol/min) (**a**) and ATP production (%) (**b**) deriving from glycolysis and oxidative phosphorylation after the sequential addition of specific inhibitors; (* *p* < 0.05 vs. WT). (**c**–**e**) Mitochondrial Stress Analysis was performed as indicated in “Materials and Methods”. Graphs represent the mean ± SD of three independent experiments of real-time oxygen consumption (OCR) rate (pmol/min/cells); (* *p* < 0.05 vs. shCTR). Mitochondrial Respiration (**c**), Basal Respiration (**d**), Maximal Respiration (**e**) were measured from OCR after the addition of specific inhibitors. (**f**–**h**) Glycolytic Stress Analysis was performed as indicated in “Materials and Methods”. Graph represents the mean ± SD of three independent experiments of Real-time extracellular acidification (ECAR) rate (mpH/min/cells); (* *p* < 0.05 vs. shCTR). Glycolitic function (**f**), Glycolysis (**g**) and Glycolytic Capacity (**h**) were measured from ECAR after the addition of specific inhibitors.

We analyzed the metabolic changes in ACC cells related to different expression levels of ERRα using Seahorse XF96 Flux Analyzer to profile oxidative phosphorylation as well as glycolysis and ATP production. The ATP Real-Time rate assay quantifies the rate of ATP production from glycolysis and mitochondria simultaneously. Data analysis revealed that shCTR, shERRα−/− and wild type H295R (WT) cells showed the same amount of ATP content. By contrast, H295R ERRα+/+ cells displayed a better performance in terms of ATP levels (Figure 2a). Moreover, in WT and shCTR cells, glycolysis and OXPHOS contributed equally to the production of ATP (Figure 2b). ERRα gene manipulation changed the energy distribution; specifically, ERRα+/+ cells are characterized by an oxidative profile, while the glycolytic rate is enhanced in ERRα−/− cells (Figure 2b). A deeper analysis by using Mito Stress assay (Figure 2c) revealed that OCR levels are increased in ERRα+/+ cells compared to shCTR and shERRα−/− cells. The most interesting aspect obtained following the inhibition of the main energy flows is that ERRα overexpression provides H295R cells with a better mitochondrial fitness in terms of basal (Figure 2d), maximal respiration rates (Figure 2e) and spare capacity (Figure S1a). In ERR−/− cells, a small but significant reduction in the maximal respiration (Figure 2e) is observed, while spare capacity reduction (Figure S1a) and basal respiration not coupled to ATP production (proton leak) significantly increased (Figure S1b).

Glycolytic functions were detected by monitoring the extracellular acidification rate (ECAR) after a sequential injection of specific inhibitors that allowed us to evaluate different glycolytic function parameters (Figure 2f). Glycolysis (Figure 2g) and glycolytic capacity (Figure 2h) were both increased in shERRα−/− and ERRα+/+ cells compared to shCTR cells, while the glycolytic reserve (Figure S1c) was increased in shERRα−/− and reduced in ERRα+/+ cells.

**Figure 3:**
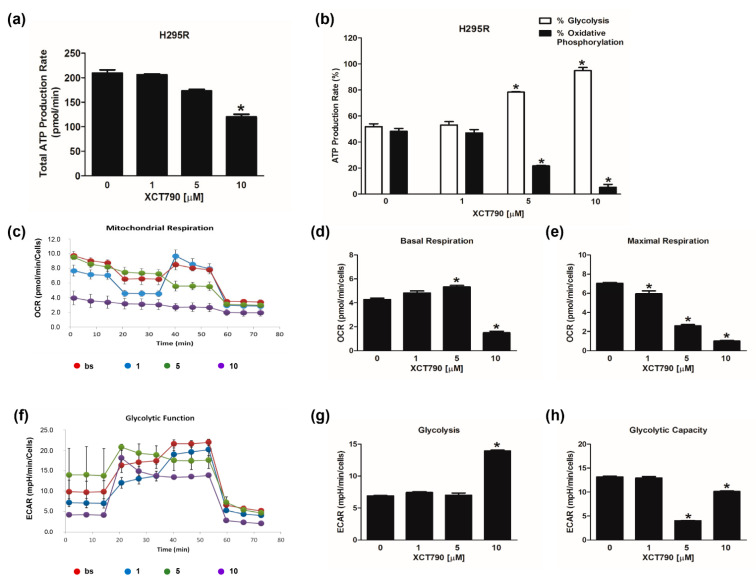
Effect of XCT790 treatment on H295R cell metabolism. The metabolic profiles of H295R cells untreated (0) or treated with XCT790 (1, 5, 10 µM) for 18h were assessed using the Seahorse XFe96 analyzer. (**a**,**b**) ATP Rate Assay was evaluated as indicated in “Materials and Methods”. Graphs represent the mean ± SD of three independent experiments of Total ATP Production Rate (pmol/min) (**a**) and ATP production (%) (**b**) derived from glycolysis and oxidative phosphorylation after the sequential addition of specific inhibitors; (* *p* < 0.05 vs. 0). (**c**–**e**) Mitochondrial Stress Analysis was performed as indicated in “Materials and Methods”. Graphs represent the mean ± SD of three independent experiments of real-time oxygen consumption (OCR) rate (pmol/min/cells); (* *p* < 0.05 vs. 0). Mitochondrial Respiration(**c**), Basal Respiration (**d**), Maximal Respiration (**e**) were measured from OCR after the addition of specific inhibitors. (**f**–**h**) Glycolytic Stress Analysis was performed as indicated in “Materials and Methods”. Graphs represent the mean ± SD of three independent experiments of real-time extracellular acidification (ECAR) rate (mpH/min/cells); (* *p* < 0.05 vs. 0). Glycolitic function (**f**), Glycolysis (**g**) and Glycolytic Capacity (**h**) were measured from ECAR after the addition of specific inhibitors.

We next investigated the effects of reduced ERRα expression on the bioenergetic functions of H295R cells by using XCT790. Results from the ATP assay showed that XCT790 lowered ATP levels, but significant effects were achieved only with the highest dose (Figure 3a). In particular, doses higher than 1 µM reduced the contribution of OXPHOS and increased the amount of ATP derived from glycolysis (Figure 3b).

**Figure 4:**
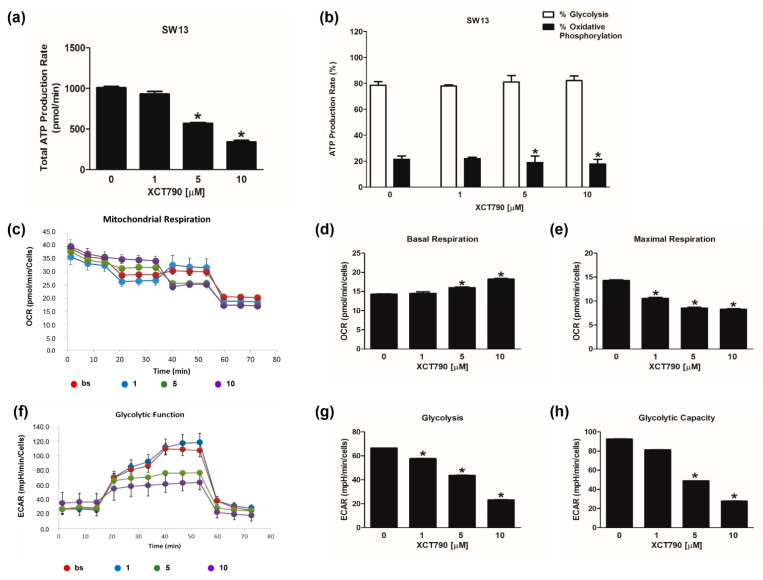
Effect of XCT790 treatment on SW13 cell metabolism. The metabolic profiles of SW13 cells untreated (0) or treated with XCT790 (1, 5, 10 µM) for 18 h were assessed by Seahorse XFe96 analyzer. (**a**,**b**) ATP Rate Assay was evaluated as indicated in “Materials and Methods”. Graphs represent the mean ± SD of three independent experiments of Total ATP Production Rate (pmol/min) (**a**) and ATP production (%) (**b**) derived from glycolysis and oxidative phosphorylation after the sequential addition of specific inhibitors (* *p* < 0.05 vs. 0). (**c**–**e**) Mitochondrial Stress Analysis was performed as indicated in “Materials and Methods”. Graphs represent the mean ± SD of three independent experiments of real-time oxygen consumption (OCR) rate (pmol/min/cells) (* *p* < 0.05 vs. 0). Mitochondrial Respiration(**c**), Basal Respiration (**d**), Maximal Respiration (**e**) were measured from OCR after the addition of specific inhibitors. (**f**–**h**) Glycolytic Stress Analysis was performed as indicated in “Materials and Methods”. Graphs represent the mean ± SD of three independent experiments of real-time extracellular acidification (ECAR) rate (mpH/min/cells); (* *p* < 0.05 vs. 0). Glycolitic function (**f**), Glycolysis (**g**) and Glycolytic Capacity (**h**) were measured from ECAR after the addition of specific inhibitors.

**Figure 5:**
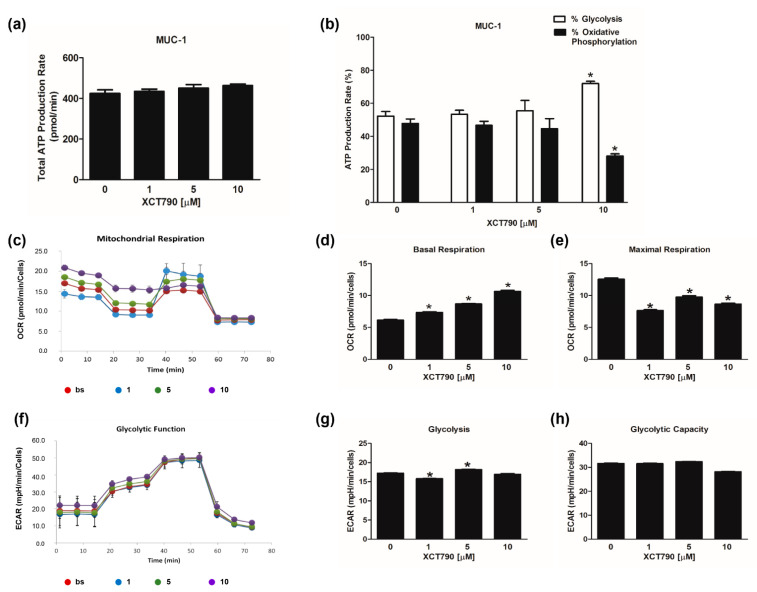
Effect of XCT790 treatment on MUC-1 cell metabolism. The metabolic profiles of MUC-1 cells untreated (0) or treated with XCT790 (1, 5, 10 µM) for 18 h were assessed by Seahorse XFe96 analyzer. (**a**,**b**) ATP Rate Assay was evaluated as indicated in “Materials and Methods”. Graphs represent the mean ± SD of three independent experiments of Total ATP Production Rate (pmol/min) (**a**) and ATP production (%) (**b**) derived from glycolysis and oxidative phosphorylation after the sequential addition of specific inhibitors (* *p* < 0.05 vs. 0). (**c**–**e**) Mitochondrial Stress Analysis was performed as indicated in “Materials and Methods”. Graphs represent the mean ± SD of three independent experiments of real-time oxygen consumption (OCR) rate (pmol/min/cells) (* *p* < 0.05 vs. 0). Mitochondrial Respiration (**c**), Basal Respiration (**d**), Maximal Respiration (**e**) were measured from OCR after the addition of specific inhibitors. (**f**–**h**) Glycolytic Stress Analysis was performed as indicated in “Materials and Methods”. Graphs represent the mean ± SD of three independent experiments of real-time extracellular acidification (ECAR) rate (mpH/min/cells); (* *p* < 0.05 vs. 0). Glycolitic function (**f**), Glycolysis (**g**) and Glycolytic Capacity (**h**) were measured from ECAR after the addition of specific inhibitors.

The evaluation of mitochondrial functions upon XCT790 treatment (Figure 3c) revealed that basal respiration rate decreased in H295R cells treated with 10 µM (Figure 3d). The maximal respiration rate (Figure 3e) and spare capacity (Figure S1d) were dose-dependently decreased by XCT790, while the trend of OCR levels associated with the proton leak was similar to those of basal respiration (Figure S1e). Glycolytic flux analysis (Figure 3f) revealed that glycolysis increased (Figure 3g) with the highest dose of XCT790 while the glycolytic capacity (Figure 3h) and reserve (Figure S1c) were down-regulated. Dose-dependent effects of XCT790 were observed on SW13 cells. Drug treatment decreased OCR and ECAR values associated with all parameters related to ATP content (Figure 4a,b), mitochondrial metabolism (Figure 4c–e and Figure S2a,b) and glycolysis (Figure 4f–h and Figure S2c). The metabolic profile of mitotane-resistant MUC-1 cells showed that total ATP content was unaffected by XCT790 (Figure 5a) but the highest dose of XCT790 caused a shift from a balanced energy state to an increased glycolytic function (Figure 5b). Accordingly, the evaluation of mitochondrial respiration (Figure 5c) showed that XCT790 treatment reduced maximal respiration (Figure 5e) and spare capacity (Figure S2d) while enhanced the basal respiration (Figure 5d) and the proton leak (Figure S2e). Moreover, the glycolysis and all glycolytic parameters were only modestly affected (Figure 5f–h and Figure S2f).

### 3.3. Changes in ERRα Expression Affect ACC Cell Motility

**Figure 6:**
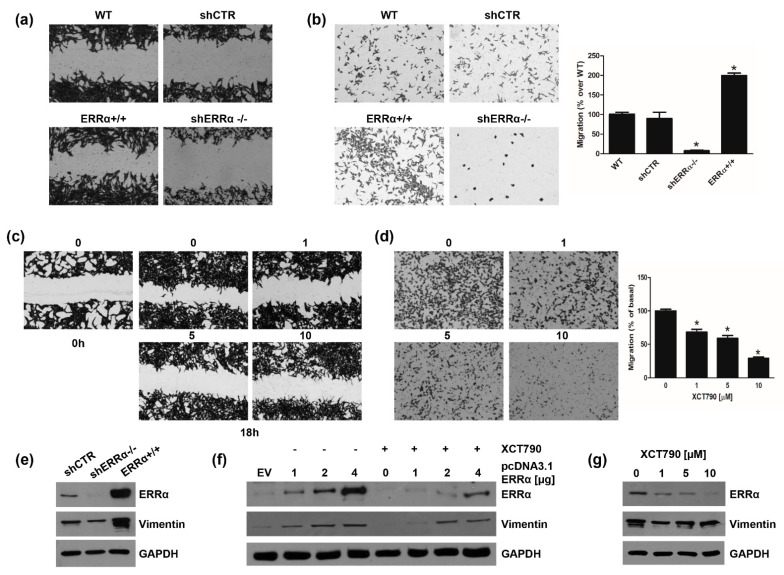
ERRα modulates H295R cell motility and Vimentin expression. (**a**,**b**) H295R (WT), H295R clones, knock in (ERRα+/+) or knock out (shERRα−/−) for ERRα gene, and H295R cell stably transfected with control plasmid (shCTR) were used in Wound Healing (**a**) and Boyden Chamber (**b**) assays as reported in “Materials and Methods”. Images are from a representative experiment. (**c**,**d**) H295R cells were treated with vehicle (0) or XCT790 (1, 5, 10 μM) for 18 h and Wound Healing (**c**) and Boyden Chamber (**d**) assays were performed as reported in “Materials and Methods”. Images are from a representative experiment. (**c**) The wounds were observed under an inverted microscope immediately (0 h) and 18 h after the scratch (100× magnification). (**b**,**d**) Migrated cells were photographed under an inverted microscope and counted (see Material and Methods), 20× magnification. Graphs represent the mean ± SD of three independent experiments. The number of untreated cells (0) was set as 100% (* *p* < 0.05 vs. 0). (**e**) Total proteins from H295R clones (shCTR, shERRα−/−, ERRα+/+) were analyzed by western Blotting (WB) using antibodies against ERRα and Vimentin. GAPDH was used as a loading control. Blots are representative of three independent experiments with similar results. (**f**) H295R were transfected for 48 h with pcDNA3.1 non containing (EV) or containing ERRα coding sequence (pcDNA3.1-ERRα). After transfection cells were left untreated (−) or treated (+) for 24 h with XCT790 (10 μM). Total proteins were analyzed by WB using antibodies against ERRα and Vimentin. GAPDH was used as a loading control. (**g**) Cells were untreated (0) or treated with XCT790 (1, 5, 10 μM) for 24 h. Total proteins were analyzed by WB using antibodies against ERRα and Vimentin. GAPDH was used as a loading control. Original image of western blot can be found at File S1.

We first assessed the motility of H295R cell clones. Scratch assay demonstrated that ERRα overexpression significantly increased H295R cell motility, which was reduced in shERRα−/− cells (Figure 6a). Similar results were obtained using Boyden Chamber assays (Figure 6b). We next investigated the effects of increasing doses of XCT790 on H295R cell motility. XCT790 exposure for 18 h (a time point not sufficient to cause cell death) decreased H295R migration in a dose dependent manner as evidenced by scratch (Figure 6c) and Boyden Chamber (Figure 6d) assays. Moreover, expression levels of Vimentin, a known EMT marker, were increased in ERRα+/+ cells and reduced in shERRα−/− (Figure 6e). These results were further confirmed in H295R cells transiently transfected with a pcDNA3.1ERRα (Figure 6f; Figure S4a). XCT790 treatment was able to reduce Vimentin expression in both H295R (WT) (Figure 6g; Figure S4b) and ERRα overexpressing cells (Figure 6f; Figure S4a).

**Figure 7:**
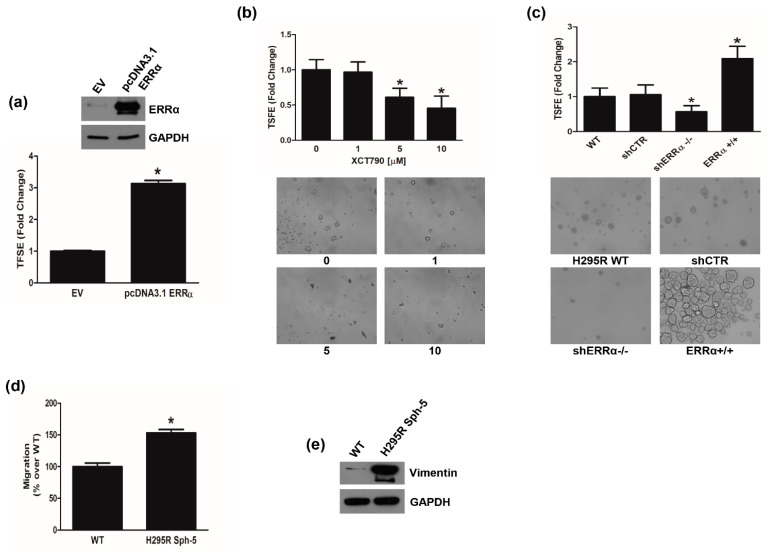
ERRα promotes H295R spheroids formation. (**a**) H295R were transfected for 48 h with pcDNA3.1 non containing (EV) or containing ERRα coding sequence (pcDNA3.1-ERRα) and then grown as 3D spheroids for 5 days. Spheroids were counted under an inverted microscope and results were expressed as fold change over control (EV) ± SD (TSFE, tumor spheroids formation efficiency); (* *p* < 0.05 vs. EV). Insert confirms ERRα overexpression. (**b**) H295R cells were left untreated (0) or treated with XCT790 (1, 5, 10 μM) for 24 h and TSFE was evaluated 5 days later (* *p* < 0.05 vs. 0). Images below graph are from a representative experiment (20× magnification). (**c**) Wild type H295R (WT) and H295R clones (shCTR, shERRα−/−, ERRα+/+) were used to evaluate 3D spheroids formation. TSFE was evaluated 5 days later (* *p* < 0.05 vs. WT). Images below graph are from a representative experiment (20× magnification). (**d**) H295R spheroids (H295R Sph-5) were allowed to grow for 5 days and then trypsinized and reseeded weekly in spheroid media for 5 weeks. Boyden Chamber Assay was performed as reported in the “Materials and Methods”. Migrated cells were randomly photographed and counted with ImageJ software (* *p* < 0.05 vs. WT). (**e**) H295R (WT) cells and H295R grown as spheroids for 5 weeks (H295R Sph-5), were analyzed by WB using antibody against Vimentin. GAPDH was used as a loading control. Blots are representative of three independent experiments with similar results. Original image of western blot can be found at File S1.

These data well correlated with the observation that transient ERRα overexpression in H295R cells enhanced the number of 3D spheroids (Figure 7a). By contrast, XCT790 treatment reduced the ability of H295R cells to grow in non-adherent conditions preventing spheroid formation (Figure 7b). These data were further confirmed in H295R clones (Figure 7c). shERRα−/− cells showed a lower efficiency to grow as spheroids compared to shCTR or wild type cells, while ERRα+/+ cells easily formed spheroids (Figure 7c). These cells manifested a greater migratory ability than adherent cells (Figure 7d) and showed an enhanced expression of Vimentin (Figure 7e).

**Figure 8:**
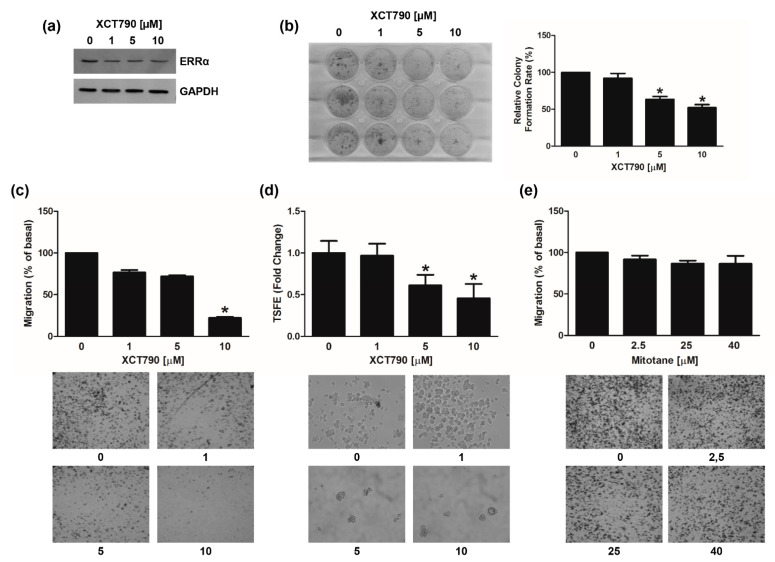
Effects of XCT790 treatment on MUC-1 cells. (**a**) MUC-1 cells were left untreated (0) or treated with XCT790 (1, 5, 10 μM) for 24 h. Total proteins were analyzed by WB using antibodies against ERRα. GAPDH was used as a loading control. Blots are from one experiment representative of three with similar results. (**b**) MUC-1 cells were seeded in 12-well plates and allowed to grow in the absence or presence of different XCT790 (1, 5, 10 μM) doses for 14 days. Colonies were stained with 0.05% Coomassie Blue in methanol/water/acetic acid (45:45:10, *v*/*v/v*). Colony number (relative colony formation rate) was assessed using Image J software and normalized to untreated cells (0). (**c**,**d**) MUC-1 cells were seeded in the Boyden insert and vehicle (0), XCT790 (1, 5, 10 μM) (C) or mitotane (2.5, 25, 40 μM) (**d**) were added in the upper chamber; cells were allowed to migrate across the membrane for 18 h. Migrated cells were photographed under an inverted microscope and counted (see Material and Methods), with 20× magnification. The number of untreated cells (0) was set as 100% (* *p* < 0.05 vs. 0). Images below are from a representative experiment (20× magnification). (**e**) MUC-1 cells were untreated (0) or treated with XCT790 (1, 5, 10 μM) for 24 h. TSFE was evaluated 5 days later. Results were expressed as fold change over untreated cells (0) ±SD; (* *p* < 0.05 vs. 0). Images are from a representative experiment (20× magnification). Original image of western blot can be found at File S1.

We also investigated the effects of XCT790 on MUC-1 and SW13 cell lines. In MUC-1 cells, the drug was effective in reducing ERRα protein expression (Figure 8a), colony formation (Figure 8b), motility (Figure 8c) and spheroid formation efficiency (Figure 8d) however to a smaller extend compared to H295R cells. A sub-therapeutic (25 µM) and a therapeutic dose (40 µM) of mitotane were tested in parallel experiments (Figure 8e) confirming MUC-1 as mitotane-resistant cells. In SW13 cells, XCT790 treatment was as effective as in H295R cells in decreasing ERRα protein expression (Figure S3a), cell viability (Figure S3b), cell motility (Figure S3c) and spheroid formation efficiency (Figure S3d).

### 3.4. Cholesterol Modulates ERRα Activity in ACC

**Figure 9:**
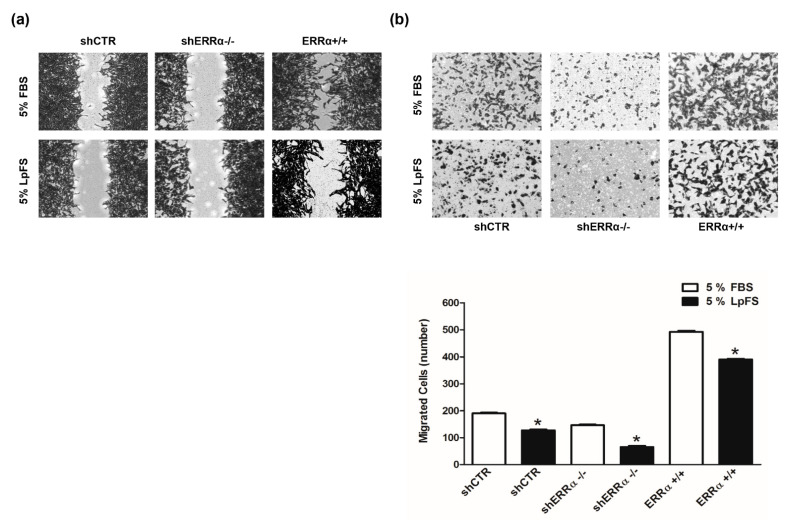
ERRα expression levels and cholesterol influence H295R cell migration. (**a**) H295R clones (shCTR, ERRα+/+, shERRα−/−) were maintained in 5% FBS or 5% LpFS containing medium. Cells were used in Wound Healing (**a**) and Boyden Chamber (**b**) assays performed as reported in “Materials and Methods”. (**a**) Images are from a representative experiment (100× magnification). (**b**) Migrated cells were photographed under an inverted microscope (20× magnification) and counted with ImageJ software. Graphs represent the mean ± SD of three independent experiments (* *p* < 0.05 vs. FBS).

It has been demonstrated that cholesterol through binding ERRα influence the metabolic pathways in breast cancer [11]. To verify a similar functional interaction between ERRα and cholesterol in ACC, H295R clones (shCTR, shERRα−/−, ERRα+/+) were maintained in medium containing FBS or lipoprotein-free serum (LpFS) to be tested in wound healing (Figure 9a) and Boyden chamber (Figure 9b) assay. Clones grown in FBS containing medium confirmed data from Figure 6a,b. The absence of cholesterol (LpFS) negatively affected motility of all tested cells. In particular, ERRα+/+ cells in LpFS manifested a reduced ability to close the wound and to migrate across membrane in Boyden chambers.

## 4. Discussion

The current study aimed to investigate the role of ERRα in ACC cell metabolism by modulating its expression and evaluating the impact on ACC progression.

In the first part of the study, we performed proteomic analysis to delineate the differential expression between untreated and XCT790-treated H295R cells. The inverse agonist of ERRα was able to significantly alter the expression of a large number of proteins. In particular, KEGG enrichment analyses identified overrepresented pathways, with the majority composed of genes involved in cell metabolism (glycolysis/gluconeogenesis, pentose phosphate pathway, oxidative phosphorylation, pyruvate metabolism, fatty acid elongation and degradation, along with others) and down-regulated by XCT790. By contrast, genes upregulated by the treatment belong to pathways involved in the protein processing in endoplasmic reticulum, apoptosis and protein degradation (proteasome). Similar results were observed in breast cancer cells [23]. According to the metabolic role of ERRα, several of these genes have been shown to be physiologically relevant ERRα targets [24] and involved in tumor biology as documented by the active research in this field [25,26,27].

Data derived from proteomic analysis were implemented by functional studies on ACC cell metabolism using Seahorse XF analyzer, which allows real-time analysis of glycolytic and mitochondrial flows. In order to dissect the impact of ERRα on ACC metabolism, we selected stable H295R clones overexpressing (ERRα+/+) or with a silenced (shERRα−/−) ESRRA gene expression. As expected, cells overexpressing ERRα displayed higher ATP content compared to shERRα−/− and shCTR cells. Specifically, ERRα+/+ cells are characterized by an oxidative profile while the glycolytic rate is enhanced in shERRα−/− cells, which is surprising considering that ERRα target genes belong to both glycolytic and mitochondrial pathways. This ability brings up different factors and/or alternative activated pathways. Indeed, the glycolytic assay revealed that although the glycolytic capacity increased both in shERRα−/− and ERRα+/+ cells, the glycolytic reserve increased only in shERRα−/− cells while it was reduced in ERRα+/+ cells. This observation suggests that, while alternative metabolic pathways are activated in response to ERRα depletion, ERRα overexpression considerably increases the oxidative metabolic pathway and favors a greater mitochondrial coupling efficiency (data not shown) to the expense of reserve capacity. Accordingly, Seahorse analyses suggested that ERRα overexpression gives a better mitochondrial fitness to H295R cells, while shERRα−/− cells have reduced basal and maximal respiration rates as well as the spare capacity. In these cells, a significant increase in the proton leak parameter was observed, indicative of defective mitochondria, explained by the role of ERRα as master regulator of cell metabolism mainly associated with mitochondria [28].

We next investigated the effects of reduced ERRα expression on the bioenergetic functions of three ACC cell lines by using XCT790. Results from the ATP assay showed that XCT790 lowered ATP levels in mitotane responsive H295R and SW13 cells, while it was ineffective in mitotane-resistant MUC-1 cells. Indeed, the analysis of the energetic contribution of mitochondria and glycolysis reveals an extreme metabolic plasticity in MUC-1 cells and H295R compared to SW13 cells, which exhibit a glycolytic phenotype. The evaluation of mitochondrial functions revealed the ability of XCT790 to negatively affect the maximal respiration rate in all three cell lines. In addition, the spare respiratory capacity was dose-dependently impaired in all cell models, indicating an effective ability of the cells to cope with sudden increased need for ATP. The mitochondrial spare capacity is an important parameter concerning the mitochondrial functions. When cells are subjected to stress, energy demand increases, and more ATP is required to sustain cellular functions. A cell with a larger spare respiratory capacity can produce more ATP and overcome more stress, which is estimative of a cell’s ability to cope with large increases in ATP turnover. Consequently, exposure to XCT790 can adversely affect the ability of cells to cope with other stresses. This observation paves the way for further studies on the potential additive effects of combined therapies in drug-resistant ACC phenotypes. Indeed, in our cell models the increased glycolytic activity, that is more pronounced in MUC-1 cells, seems to be the main adaptive metabolic response under XCT790 stress.

ERRα, with its dual role as metabolic gatekeeper and transcription factor, has a great impact on tumor progression, since it drives the expression of many genes involved in invasion, angiogenesis and metastasis in several tumors [9]. Accordingly, the absence of ERRα is able to impair tumorigenic potential in aggressive xenografted breast cancer cells where the ERRα/PGC-1α complex binds to a VEGF promoter region regulating its expression, and promoting tumor angiogenesis and invasion. In addition, ERRα knockdown attenuated the migration and invasion processes of endometrial cancer cells [29], gastric cancer [26], non-small cell lung carcinoma [30] and bladder cancer [31].

Our data clearly revealed a direct impact of ERRα expression on H295R cell motility. ERRα overexpression significantly increased H295R cell migration and expression of the EMT marker Vimentin, that were decreased by down-regulation of the metabolic receptor, by either genetic ablation or by pharmacological intervention.

ERRα involvement in ACC aggressiveness is further supported by its influence on H295R cell’s ability to grow in non-adherent conditions as 3D spheroids, a feature that characterizes tumor-initiating stem-like cells (TICs). TICs are a small sub-population of tumor cells resistant to most anti-cancer therapies which share many features with stem cells [23]. XCT790 was able to reduce 3D spheroids formation and motility not only in H295R cells, but also in SW13 cells and, above all, in mitotane-resistant MUC-1 cells suggesting, once again, that targeting ERRα could be an effective therapy for the treatment of mitotane-resistant ACC phenotype. Interestingly, long serial 3D spheroid culture (H295R Sph-5) showed enhanced motility and Vimentin expression compared to H295R cells grown in adherent conditions. We are currently working to define the metabolic changes associated with this more aggressive phenotype.

Our experiments also revealed that motility assays performed in H295R cells were negatively affected by lipoprotein-deprived serum, thus without cholesterol, confirming the steroid as an ERRα activator. It is therefore evident that ERRα plays a dual role, as an important metabolic adaptive regulator and as a transcriptional modulator of genes involved in different energy-intensive processes promoting tumor progression such as EMT. Accordingly, in ovarian cancer cell lines, ERRα down-regulation reduced mitochondrial activity avoiding EMT and migration [32]. Our previous study [33] demonstrated that in ACC cells, ERRα protein depletion by XCT790 caused a reduction in mitochondrial mass and function leading to cell death. Accordingly, in vivo experiments with H295R xenografts confirmed that pharmacological inhibition of ERRα strongly inhibited ACC cell growth without exerting any marked toxic effect. Our results are supported by additional in vivo studies performed with breast [34], endometrial [35] and pancreatic [36] cancer cells, which altogether point to ERRα as a specific target for the treatment of high energy demanding cells such as tumor cells.

## 5. Conclusions

Our findings highlight ERRα as a key regulator of ACC metabolism related to cell motility. Targeting this receptor has the potential to strongly inhibit the growth of mitotane-sensitive and −resistant ACC cells and prevent the transition of ACC cells to a more aggressive phenotype. For these reasons, ERRα can be considered a relevant target to be included in the search for new therapeutic agents to fight ACC growth and progression.

## Data Availability

The data presented in this study are available on request from the corresponding author.

## Supplementary Materials

The following are available online at https://www.mdpi.com/article/10.3390/cancers14163885/s1, Figure S1: Metabolic changes in H295R cells related to different expression levels of ERRα. The metabolic profiles of H295R shCTR, shERRα−/−, ERRα+/+ cells (a,b) and H295R untreated (0) or treated with XCT790 (1, 5, 10 μM) for 18h (d,e), were assessed using the Seahorse XF-e96 analyzer. (a,b,d,e) Mitochondrial Stress Analysis was performed as indicated in “Materials and Methods”. Graphs represent the mean ± SD of three independent experiments of Real-time oxygen consumption (OCR) rate (pmol/min/cells); (* *p* < 0.05 vs. shCTR). Spare Capacity (a,d), Proton Leak (b,e), were measured from OCR after the addition of specific inhibitors. (c,f) Glycolytic Stress Analysis was performed as indicated in “Materials and Methods”. Graph represents the mean ± SD of three independent experiments of Real-time extracellular acidification (ECAR) rate (mpH/min/cells); (* *p* < 0.05 vs. 0). Glycolytic Reserve was measured from ECAR after the addition of specific inhibitors. Figure S2. Effect of XCT790 treatment on SW13 and MUC-1 cell metabolism. The metabolic profiles of SW13 (a–c) and MUC-1 (d–f) cells untreated (0) or treated with XCT790 (1, 5, 10 µM) for 18 h were assessed using the Seahorse XF-e96 analyzer. (a,b,d,e) Mitochondrial Stress Analysis was performed as indicated in “Materials and Methods”. Graphs represent the mean ± SD of three independent experiments of Real-time oxygen consumption (OCR) rate (pmol/min/cells); (* *p* < 0.05 vs. 0). Spare Capacity (a,d), Proton Leak (b,e), were measured from OCR after the addition of specific inhibitors. (c,f) Glycolytic Stress Analysis was performed as indicated in “Materials and Methods”. Graph represents the mean ± SD of three independent experiments of Real-time extracellular acidification (ECAR) rate (mpH/min/cells); (* *p* < 0.05 vs. 0). Glycolytic Reserve was measured from ECAR after the addition of specific inhibitors. Figure S3. Effect of XCT790 on ERRα protein expression, cell viability and motility in SW13 cells. SW13 cells were untreated (0) or treated with XCT790 (1, 5, 10 μM) for 24 h (a) or 18 h (c) or different times (24, 48, 72, 96 h) (b). (a) Total proteins were analyzed by WB using antibodies against ERRα. Blots are from one experiment representative of three with similar results. GAPDH was used as loading control. (b) Cell viability was evaluated by MTT assay; (* *p* < 0.05 vs. 0). (c) In the Boyden Chamber Assay, migrated cells to the lower surfaces of the membranes were observed under an inverted microscope and then counted; 20× magnification. Graph represents the mean ± SD of three independent experiments of migrated cells number expressed setting untreated cells (0) as 100%; (* *p* < 0.05 vs. 0). Images below are from a representative experiment (20× magnification). (d) TSFE was evaluated 5 days later; (* *p* < 0.05 vs. 0). Results were expressed as fold change over untreated cells (0) ±SD; (* *p* < 0.05 vs. 0). Images below are from a representative experiment (20X magnification). Figure S4. Densitometric analysis of Vimentin expression. (a) Densitometric analysis of Vimentin expression of H295R transfected for 48 h with pcDNA3.1 non containing (EV) or containing ERRα coding sequence (pcDNA3.1-ERRα). After transfection cells were left untreated (−) or treated (+) for 24 h with XCT790 (10 μM). GAPDH was used as loading control. (b) Densitometric analysis of Vimentin expression of H295R cells untreated (0) or treated for 24 h with XCT790 (1, 5, 10 μM) GAPDH was used as loading control. The band intensities were analyzed by NIH ImageJ software. Histograms represent the mean ± SD of three independent experiments; * *p* < 0.05. File S1: Original image of western blot.

## References

[1] Jouinot A., Bertherat J. (2018). Management of endocrine disease: Adrenocortical carcinoma: Differentiating the good from the poor prognosis tumors. Eur. J. Endocrinol..

[2] Kiesewetter B., Riss P., Scheuba C., Mazal P., Kretschmer-Chott E., Haug A., Raderer M. (2021). Management of adrenocortical carcinoma: Are we making progress?. Ther. Adv. Med. Oncol..

[3] Barlaskar F.M., Hammer G.D. (2007). The molecular genetics of adrenocortical carcinoma. Rev. Endocr. Metab. Disord..

[4] Zheng S., Cherniack A.D., Dewal N., Moffitt R.A., Danilova L., Murray B.A., Lerario A.M., Else T., Knijnenburg T.A., Ciriello G. (2016). Comprehensive pan-genomic characterization of adrenocortical carcinoma. Cancer Cell.

[5] Vatrano S., Volante M., Duregon E., Giorcelli J., Izzo S., Rapa I., Votta A., Germano A., Scagliotti G., Berruti A. (2018). Detailed genomic characterization identifies high heterogeneity and histotype-specific genomic profiles in adrenocortical carcinomas. Mod. Pathol..

[6] Pinheiro C., Granja S., Longatto-Filho A., Faria A.M., Fragoso M.C., Lovisolo S.M., Lerario A.M., Almeida M.Q., Baltazar F., Zerbini M.C. (2015). Metabolic reprogramming: A new relevant pathway in adult adrenocortical tumors. Oncotarget.

[7] Deblois G., St-Pierre J., Giguere V. (2013). The PGC-1/ERR signaling axis in cancer. Oncogene.

[8] Chang C.Y., McDonnell D.P. (2012). Molecular pathways: The metabolic regulator estrogen-related receptor α as a therapeutic target in cancer. Clin. Cancer Res..

[9] Casaburi I., Chimento A., De Luca A., Nocito M., Sculco S., Avena P., Trotta F., Rago V., Sirianni R., Pezzi V. (2018). Cholesterol as an endogenous ERRα agonist: A new perspective to cancer treatment. Front. Endocrinol..

[10] Wei W., Schwaid A.G., Wang X., Wang X., Chen S., Chu Q., Saghatelian A., Wan Y. (2016). Ligand activation of ERRα by cholesterol mediates statin and bisphosphonate effects. Cell Metab..

[11] Ghanbari F., Mader S., Philip A. (2020). Cholesterol as an endogenous ligand of ERRα promotes ERRα-mediated cellular proliferation and metabolic target gene expression in breast cancer cells. Cells.

[12] Suzuki T., Miki Y., Moriya T., Shimada N., Ishida T., Hirakawa H., Ohuchi N., Sasano H. (2004). Estrogen-related receptor α in human breast carcinoma as a potent prognostic factor. Cancer Res..

[13] Fujimoto J., Alam S.M., Jahan I., Sato E., Sakaguchi H., Tamaya T. (2007). Clinical implication of estrogen-related receptor (ERR) expression in ovarian cancers. J. Steroid Biochem. Mol. Biol..

[14] Fujimura T., Takahashi S., Urano T., Kumagai J., Ogushi T., Horie-Inoue K., Ouchi Y., Kitamura T., Muramatsu M., Inoue S. (2007). Increased expression of estrogen-related receptor α (ERRα) is a negative prognostic predictor in human prostate cancer. Int. J. Cancer.

[15] Bernatchez G., Giroux V., Lassalle T., Carpentier A.C., Rivard N., Carrier J.C. (2013). ERRα metabolic nuclear receptor controls growth of colon cancer cells. Carcinogenesis.

[16] Deblois G., Giguere V. (2013). Oestrogen-related receptors in breast cancer: Control of cellular metabolism and beyond. Nat. Rev. Cancer.

[17] LeBleu V.S., O’Connell J.T., Gonzalez Herrera K.N., Wikman H., Pantel K., Haigis M.C., de Carvalho F.M., Damascena A., Domingos Chinen L.T., Rocha R.M. (2014). PGC-1α mediates mitochondrial biogenesis and oxidative phosphorylation in cancer cells to promote metastasis. Nat. Cell Biol..

[18] Bergers G., Fendt S.M. (2021). The metabolism of cancer cells during metastasis. Nat. Rev. Cancer.

[19] Hantel C., Shapiro I., Poli G., Chiapponi C., Bidlingmaier M., Reincke M., Luconi M., Jung S., Beuschlein F. (2016). Targeting heterogeneity of adrenocortical carcinoma: Evaluation and extension of preclinical tumor models to improve clinical translation. Oncotarget.

[20] Sirianni R., Chimento A., Malivindi R., Mazzitelli I., Ando S., Pezzi V. (2007). Insulin-like growth factor-I, regulating aromatase expression through steroidogenic factor 1, supports estrogen-dependent tumor Leydig cell proliferation. Cancer Res..

[21] Chimento A., Sirianni R., Casaburi I., Zolea F., Rizza P., Avena P., Malivindi R., De Luca A., Campana C., Martire E. (2015). GPER agonist G-1 decreases adrenocortical carcinoma (ACC) cell growth in vitro and in vivo. Oncotarget.

[22] Shaw F.L., Harrison H., Spence K., Ablett M.P., Simões B.M., Farnie G., Clarke R.B. (2012). A detailed mammosphere assay protocol for the quantification of breast stem cell activity. J. Mammary Gland Biol. Neoplas..

[23] De Luca A., Fiorillo M., Peiris-Pages M., Ozsvari B., Smith D.L., Sanchez-Alvarez R., Martinez-Outschoorn U.E., Cappello A.R., Pezzi V., Lisanti M.P. (2015). Mitochondrial biogenesis is required for the anchorage-independent survival and propagation of stem-like cancer cells. Oncotarget.

[24] Deblois G., Hall J.A., Perry M.C., Laganiere J., Ghahremani M., Park M., Hallett M., Giguere V. (2009). Genome-wide identification of direct target genes implicates estrogen-related receptor α as a determinant of breast cancer heterogeneity. Cancer Res..

[25] Manna S., Bostner J., Sun Y., Miller L.D., Alayev A., Schwartz N.S., Lager E., Fornander T., Nordenskjold B., Yu J.J. (2016). ERRα is a marker of tamoxifen response and survival in triple-negative breast cancer. Clin. Cancer Res..

[26] Zhong Y., He K., Shi L., Chen L., Zhou B., Ma R., Yu H., Zhang J., Shuai Y., Fei Y. (2021). Down-regulation of estrogen-related receptor α (ERRα) inhibits gastric cancer cell migration and invasion in vitro and in vivo. Aging.

[27] Park S., Safi R., Liu X., Baldi R., Liu W., Liu J., Locasale J.W., Chang C.Y., McDonnell D.P. (2019). Inhibition of ERRα prevents mitochondrial pyruvate uptake exposing NADPH-generating pathways as targetable vulnerabilities in breast cancer. Cell Rep..

[28] Audet-Walsh E., Giguere V. (2015). The multiple universes of estrogen-related receptor α and gamma in metabolic control and related diseases. Acta Pharmacol. Sin..

[29] Huang X., Wang X., Shang J., Zhaang Z., Cui B., Lin Y., Yang Y., Song Y., Yu S., Xia J. (2018). Estrogen related receptor α triggers the migration and invasion of endometrial cancer cells via up regulation of TGFB1. Cell Adhes. Migr..

[30] Mukherjee T.K., Malik P., Hoidal J.R. (2021). The emerging role of estrogen related receptorα in complications of non-small cell lung cancers. Oncol. Lett..

[31] Ye X., Guo J., Zhang H., Meng Q., Ma Y., Lin R., Yi X., Lu H., Bai X., Cheng J. (2019). The enhanced expression of estrogen-related receptor α in human bladder cancer tissues and the effects of estrogen-related receptor α knockdown on bladder cancer cells. J. Cell Biochem..

[32] Wang C.W., Hsu W.H., Tai C.J. (2017). Antimetastatic effects of cordycepin mediated by the inhibition of mitochondrial activity and estrogen-related receptor α in human ovarian carcinoma cells. Oncotarget.

[33] Casaburi I., Avena P., De Luca A., Chimento A., Sirianni R., Malivindi R., Rago V., Fiorillo M., Domanico F., Campana C. (2015). Estrogen related receptor α (ERRα) a promising target for the therapy of adrenocortical carcinoma (ACC). Oncotarget.

[34] Chisamore M.J., Wilkinson H.A., Flores O., Chen J.D. (2009). Estrogen-related receptor-α antagonist inhibits both estrogen receptor-positive and estrogen receptor-negative breast tumor growth in mouse xenografts. Mol. Cancer Ther..

[35] Kokabu T., Mori T., Matsushima H., Yoriki K., Kataoka H., Tarumi Y., Kitawaki J. (2019). Antitumor effect of XCT790, an ERRα inverse agonist, on ERα-negative endometrial cancer cells. Cell. Oncol..

[36] Liu S.L., Liang H.B., Yang Z.Y., Cai C., Wu Z.Y., Wu X.S., Dong P., Li M.L., Zheng L., Gong W. (2022). Gemcitabine and XCT790, an ERRα inverse agonist, display a synergistic anticancer effect in pancreatic cancer. Int. J. Med. Sci..

