# Supplementary material for: Estrogen Related Receptor Alpha (ERRα) a Bridge between Metabolism and Adrenocortical Cancer Progression
Source: Cancers (Basel). 2022 Aug 11;14(16):3885. doi: 10.3390/cancers14163885 (PMC9406166; doi:10.3390/cancers14163885)

Original image of western blot for Figure 6

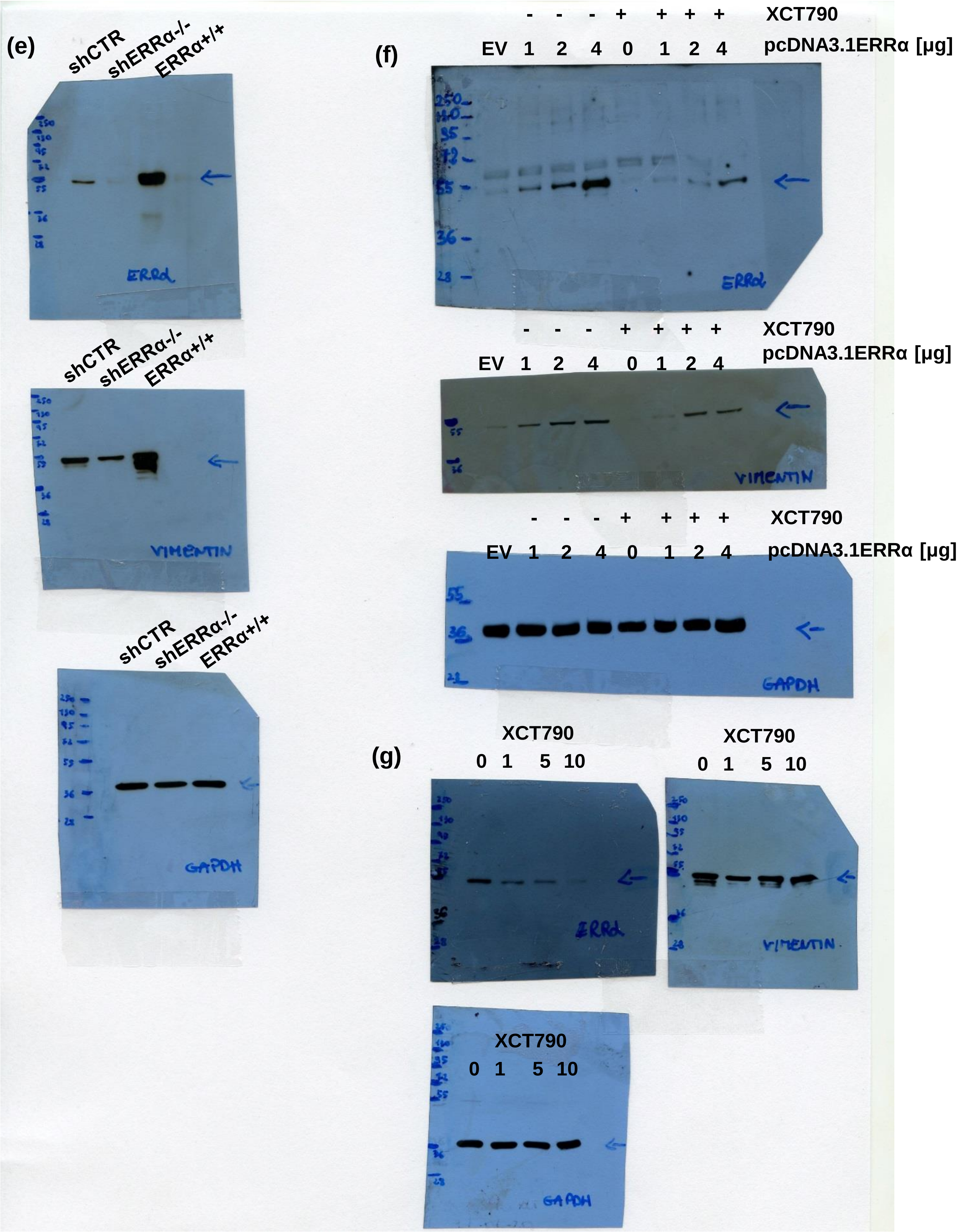

Original image of western blot for Figure 7

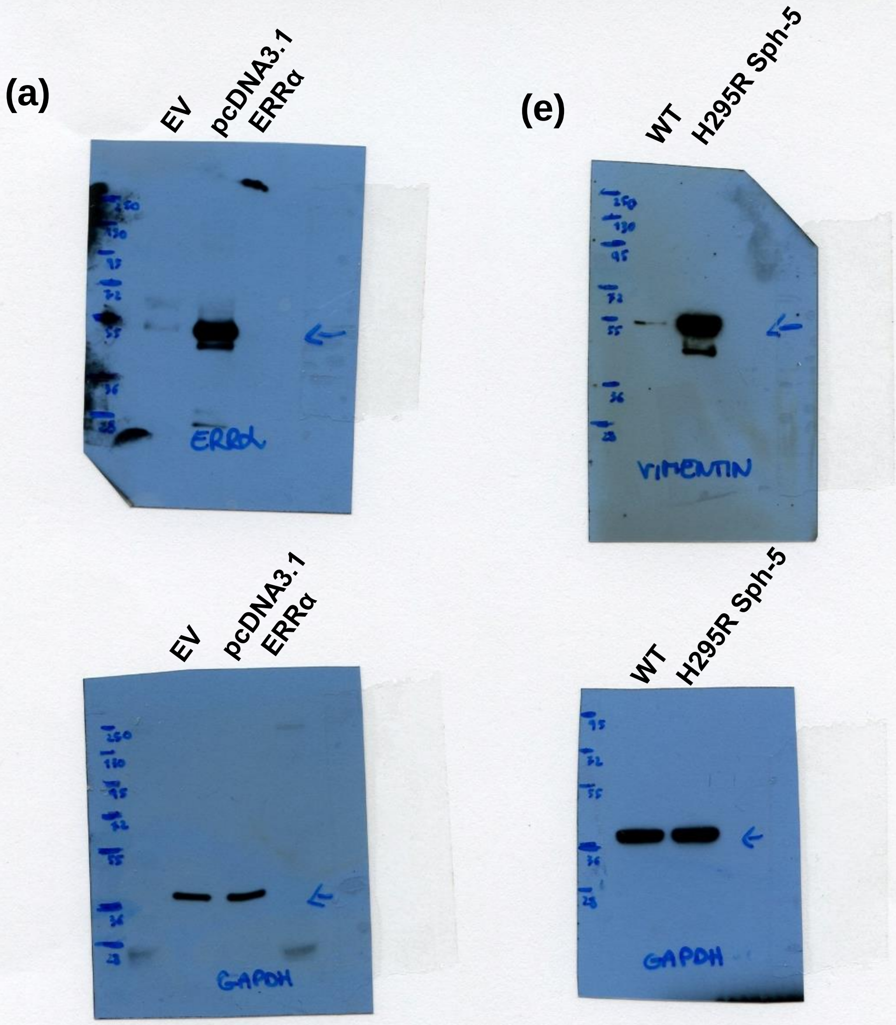

Original image of western blot for Figure 8

XCT790 [μM]

(a) 0 1 5 10

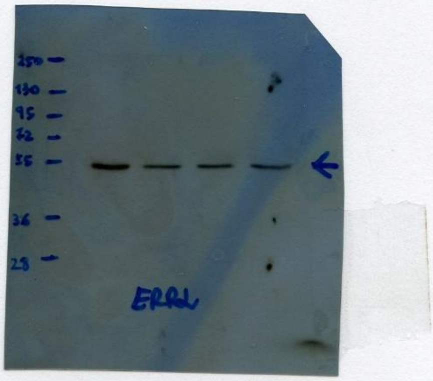

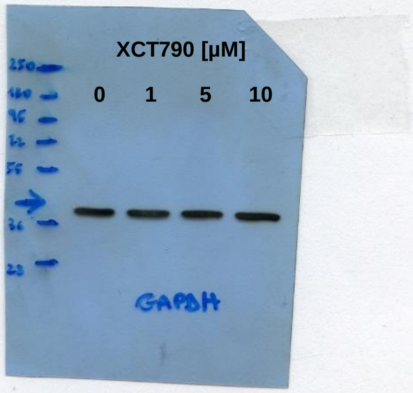

Original image of western blot for Figure S3

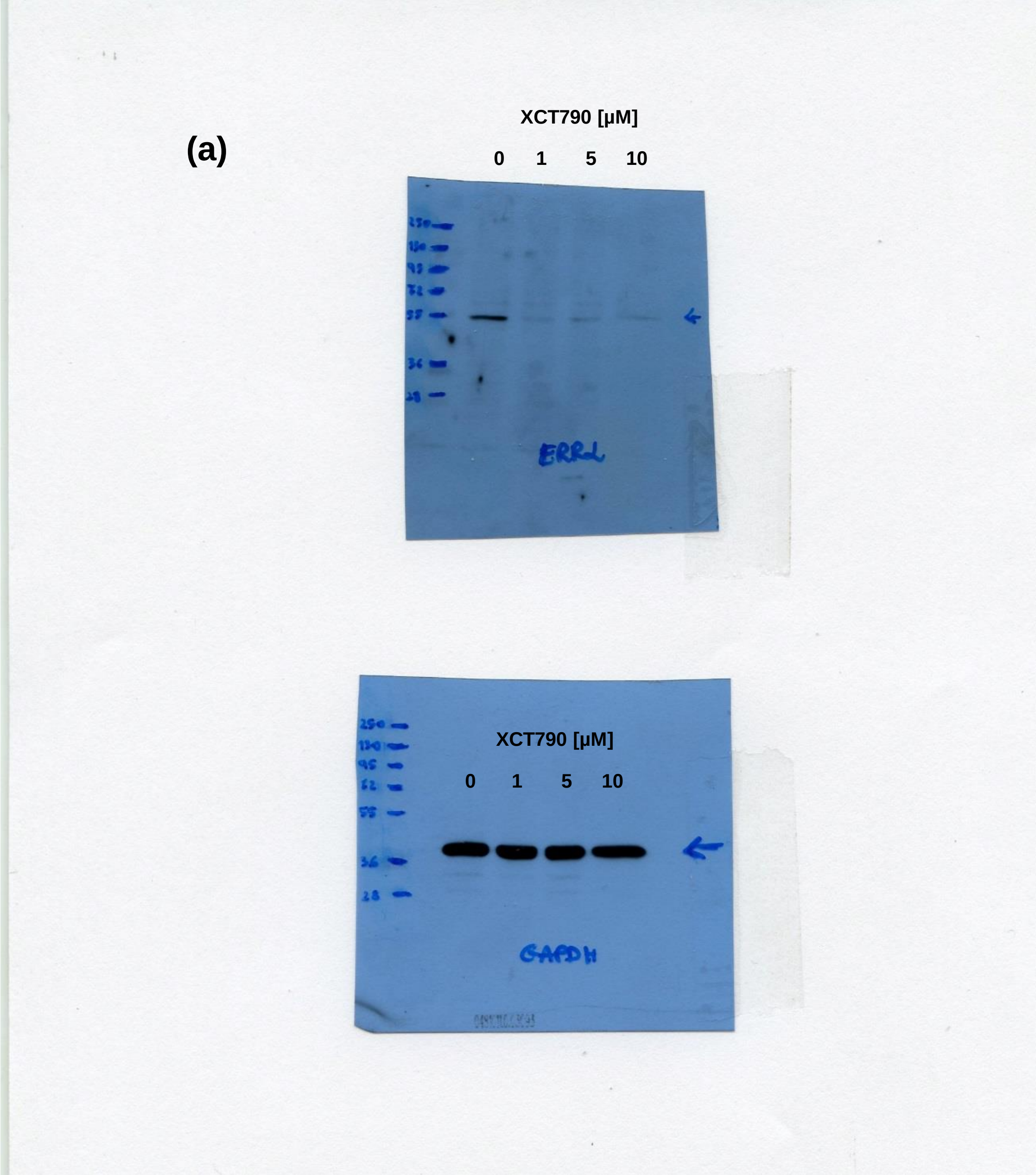

Supplement: Supplementary file 1 [file cancers-14-03885-s001.zip › cancers-1797644-supplementray File S1.pdf]
