# Supplementary figures and images for: Estrogen Related Receptor Alpha (ERRα) a Bridge between Metabolism and Adrenocortical Cancer Progression
Source: Cancers (Basel). 2022 Aug 11;14(16):3885. doi: 10.3390/cancers14163885 (PMC9406166; doi:10.3390/cancers14163885)

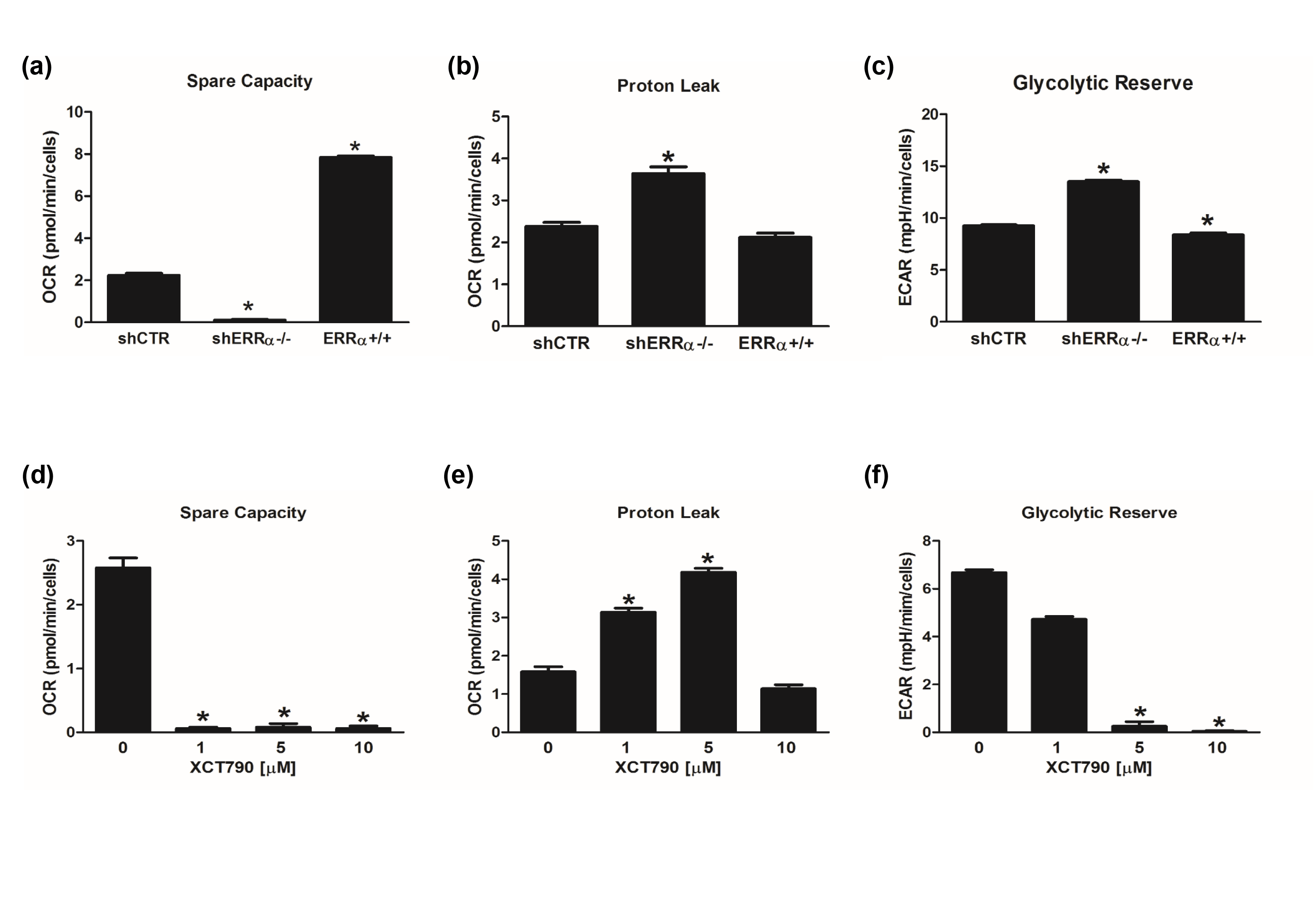

Supplement: Supplementary file 1 [file cancers-14-03885-s001.zip › Figure S1.tif]

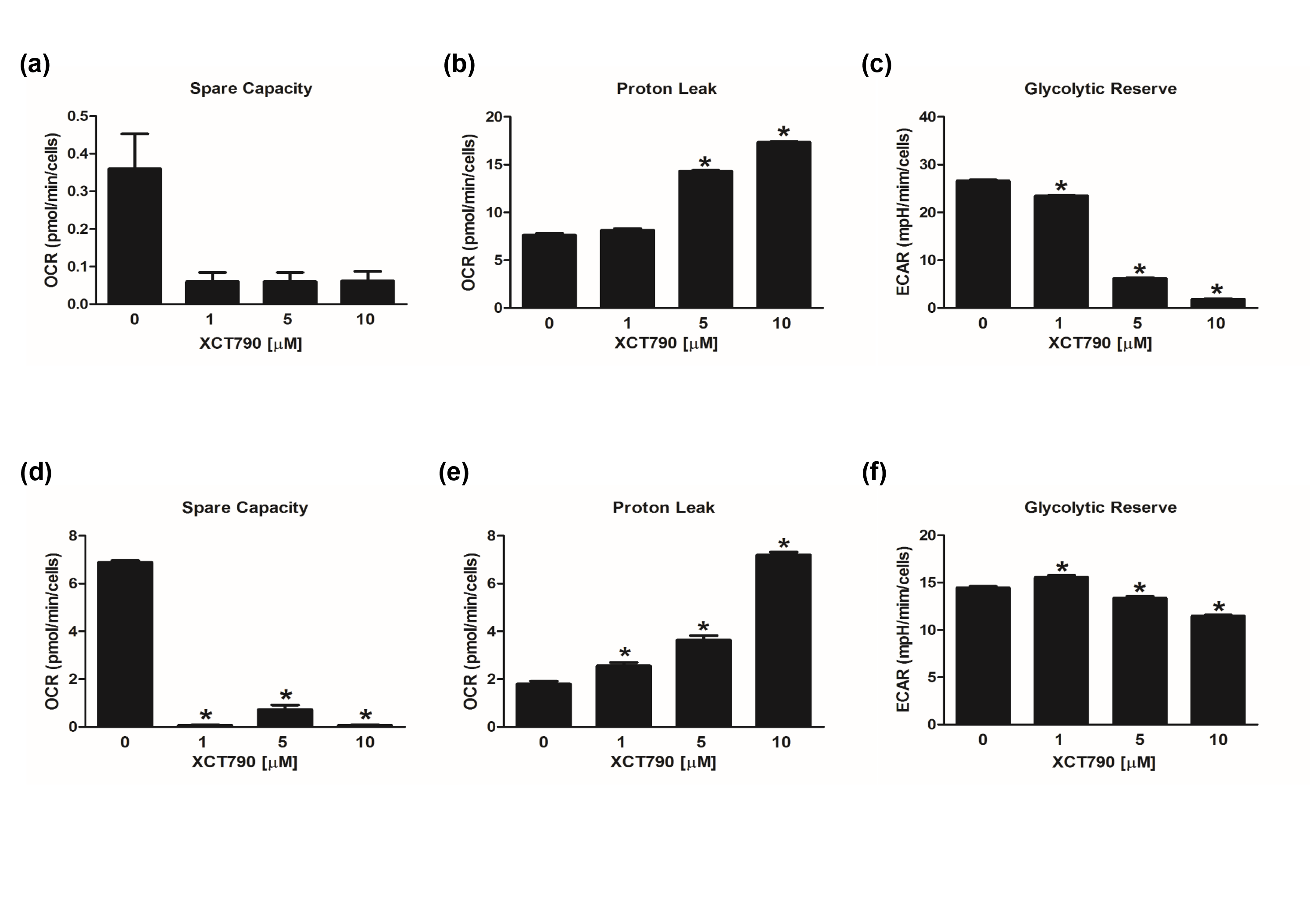

Supplement: Supplementary file 1 [file cancers-14-03885-s001.zip › Figure S2.tif]

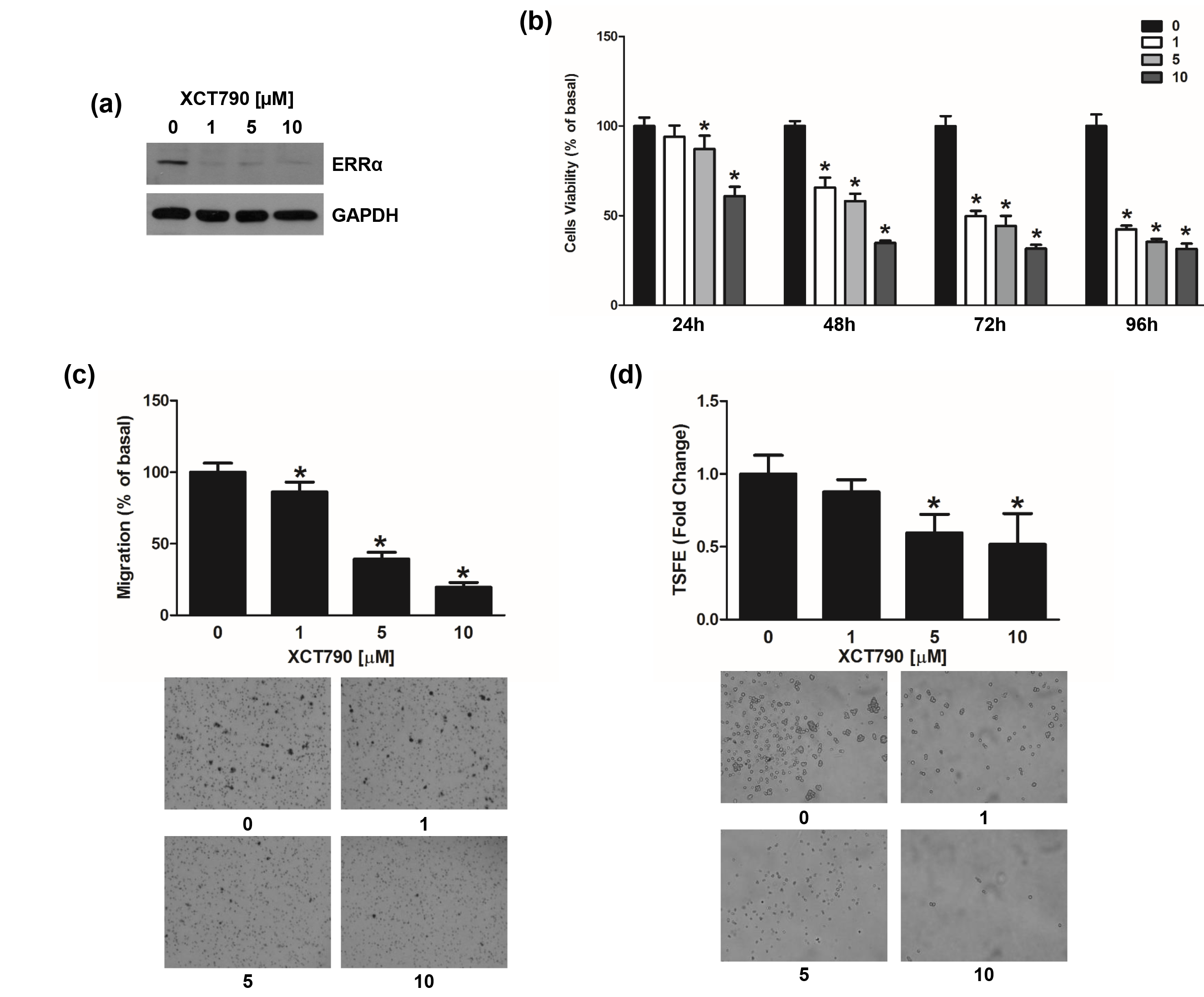

Supplement: Supplementary file 1 [file cancers-14-03885-s001.zip › Figure S3.tif]

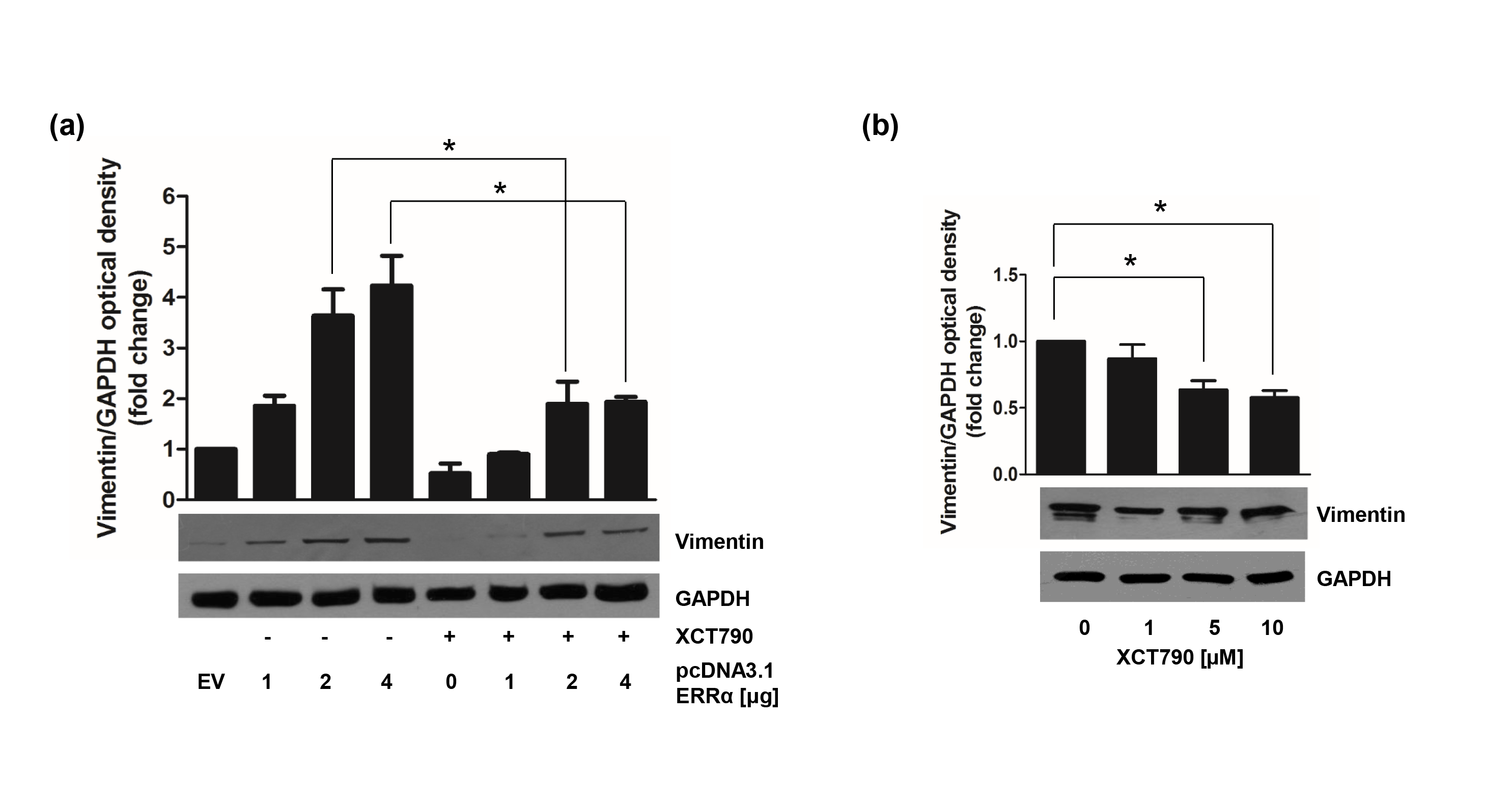

Supplement: Supplementary file 1 [file cancers-14-03885-s001.zip › Figure S4.tif]
